# Connecting Mechanism for Artificial Blood Vessels with High Biocompatibility

**DOI:** 10.3390/mi10070429

**Published:** 2019-06-28

**Authors:** Ai Watanabe, Norihisa Miki

**Affiliations:** Department of Mechanical Engineering, Keio university, 3-14-1 Hiyoshi, Kohoku-ku, Yokohama, Kanagawa 223-8522, Japan

**Keywords:** connector, artificial blood vessel, medical device, blood coagulation, implant, artificial kidney, biocompatible

## Abstract

This paper proposes a connecting mechanism for artificial vessels, which can be attached/detached with ease and does not deteriorate the biocompatibility of the vessels at the joint. The inner surface of the artificial vessels is designed to have high biocompatibility. In order to make the best of the property, the proposed connecting mechanism contacts and fixes the two artificial vessels whose contacting edges are turned inside out. In this manner, blood flowing inside the vessels only has contact with the biocompatible surface. The biocompatibility, or biofouling at the joint was investigated after in vitro blood circulation tests for 72 h with scanning electron microscopy. Blood coagulation for a short term (120 min) was evaluated by activated partial thromboplastin time (APTT). A decrease of APTT was observed, although it was too small to conclude that the connector augmented the blood coagulation. The micro dialysis device which our group has developed as the artificial kidney was inserted into the blood circulation system with the connector. Decrease of APTT was similarly small. These experiments verified that the proposed connector can be readily applicable for implantable medical devices.

## 1. Introduction

Micro/nano technologies have enabled miniaturized medical devices [1,2,3,4,5]. When they are small enough to be implanted, they can monitor and/or treat the patients continuously without bothering their daily lives. Our group has been developing micro filtering devices which we aim to use as an artificial kidney [6,7,8]. Currently, there are over 320,000 hemodialysis patients in Japan and 2.6 million patients in the world [9,10]. The hemodialysis therapy is well developed, particularly in Japan, however it leads to low quality of life of the patients. The patients are mandated to visit hospital three times a week where they receive the treatment for 4 h. Frequent punctures do not only give pain to the patients but also damage the blood vessels. The patients have severe restriction in water and salt intake. Implantable artificial kidneys will alleviate these problems and drastically improve the quality of life of patients.

**Figure 1:**
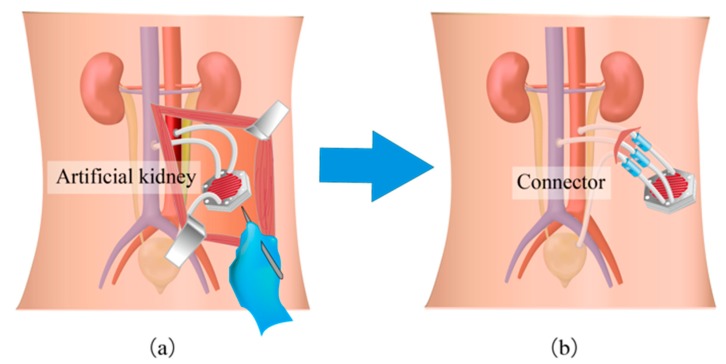
Maintenance surgery of the artificial kidney (**a**) without and (**b**) with the connecting system. It allows the device to be replaced while the connections between the artificial vessels and the artery, vein, and bladder are maintained. The surgery can be less invasive and easier by implanting the device at the shallow region beneath the skin.

One of the major challenges of the artificial kidney is biofouling, which deteriorates the dialysis performance and may mandate the device to be replaced. In order to simplify the surgery procedures and alleviate invasiveness to the patients, we propose to use a connector, as shown in Figure 1. The device is connected to the blood vessels with the biocompatible artificial vessels, which can be separated at the connector. In replacement of the device, or maintenance surgery, the access to the blood vessels is preserved and only the device is exchanged. When the device is implanted at the shallow region beneath the skin, the surgery will be of further ease.

The requirements for the connector include high biocompatibility, as well as ease of manipulation. In this paper, we propose a connector mechanism which makes the best of the high biocompatibility of the artificial vessels [11,12,13]. The connector brings the artificial vessels in contact, with their edges being turned inside out, so that blood only comes in contact with the highly biocompatible inner surface of the artificial vessels. It has a snap-fit mechanism for simple and firm connection. The detailed mechanism was discussed and its biocompatibility, or blood coagulability, of the connector was experimentally assessed. The proposed connector mechanism can be readily applicable to other implantable medical devices.

## 2. Design and Assembly of the Connector

### 2.1. Biocompatibility

The biocompatibility that we focus on in this work is blood anti-coagulation. Blood coagulation leads to the formation of blood clots and the clogging of the medical device. In case of the artificial kidney, its filtration capacity deteriorates with the coagulation. One of the major factors of blood coagulation is the contact of blood to foreign body surfaces [14]. Another factor is turbulence in the blood flow which can be caused by the geometry of the flow paths [15,16,17]. The proposed connector can be the solution for both factors. The blood anti-coagulation can be assessed by optical investigation of thrombus at the surface and activated partial thromboplastin time (APTT), which represents the speed of blood coagulation [17,18].

### 2.2. Mechanism Design and Assembly

In order to exploit the high biocompatibility of the artificial blood vessels, the connector is designed such that blood only contacts the inner surfaces of the artificial blood vessels to be connected.

**Figure 2:**
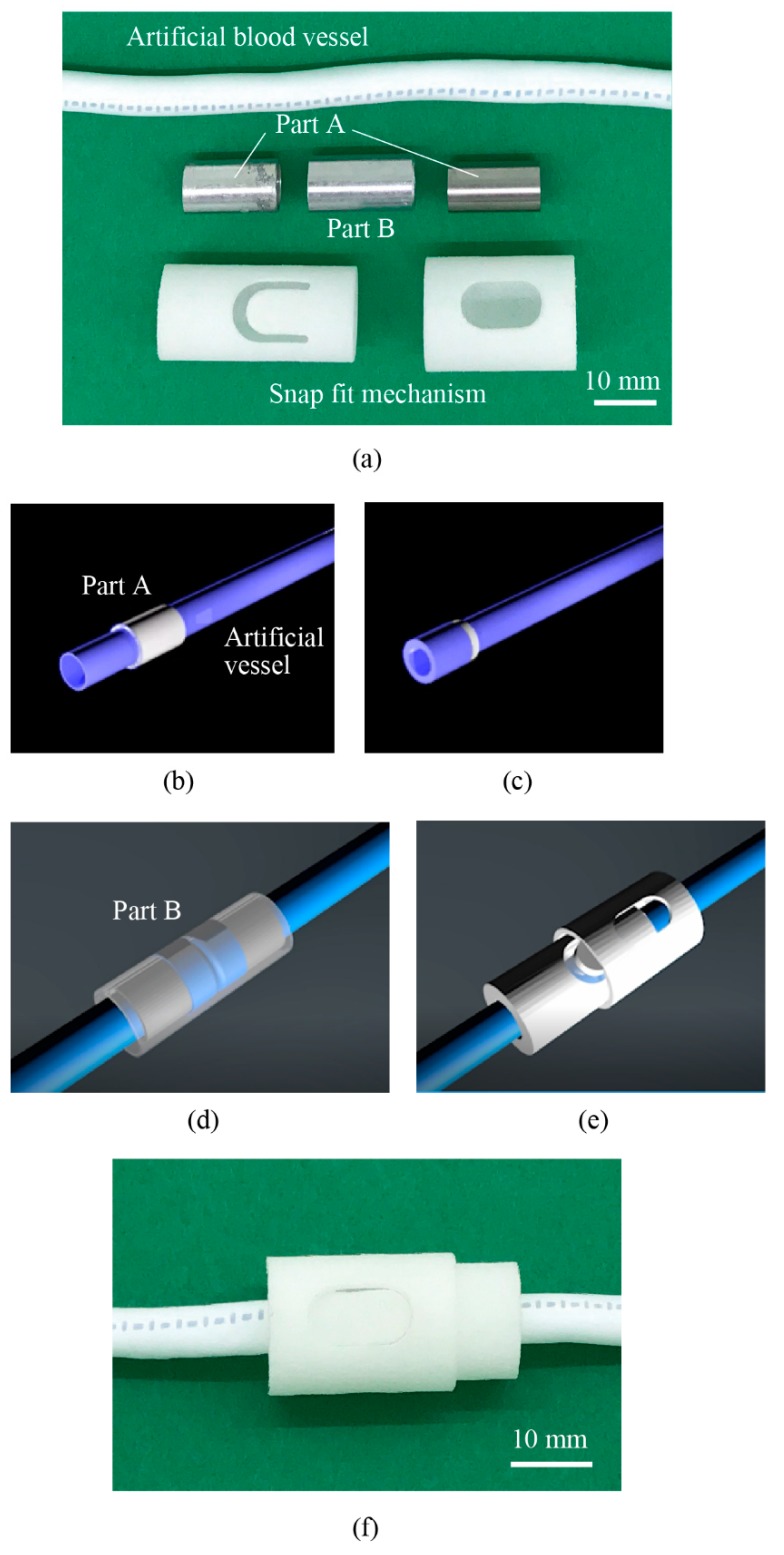
Proposed connecting mechanism of artificial blood vessels. (**a**) The mechanism consists of metal part A and B, 3D-printed snap fit mechanism, and artificial vessels. (**b**,**c**) The edges of the vessels to be joined are turned inside out over part A. (**d**) They are brought into contact inside part B. (**e**) The snap fit mechanism secures the connection. (**f**) The photo of the connected mechanism. The resulting size of the connection mechanism is 15.4 mm in diameter and 29.0 mm in length.

The connector consists of the cylindrical parts A and B made of metal and the snap-fit mechanism that was manufactured by 3D printing, as shown in Figure 2a. The artificial vessels are the ePTF graft (thin-wall straight type, W. L. Gore & Associates, Co., Ltd., Tokyo, Japan). The inner and outer diameter are 6.0 mm and 6.8 mm, respectively. The parts A and B are manufactured from stainless steel (SUS304) with inner and outer diameters of 6.8 mm and 7.6 mm and 8.2 mm and 9.0 mm, respectively. The outermost parts for the snap-fit mechanism are 3D-printed from Nylon 12.

**Figure 3:**
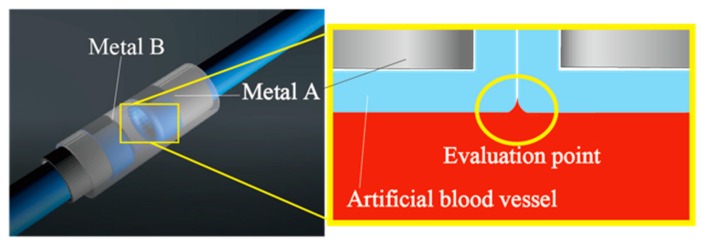
Joint area inside the connector. A small ditch is formed at the interface, where blood coagulation might be promoted.

First, the artificial vessels are inserted into the cylindrical part A and the edges to be contacted are turned inside out (Figure 2b,c shows the case for one artificial vessel). They are brought into contact and the cylindrical part B covers the joint part, as shown in Figure 2d. The 3D-printed snap-fit mechanism is brought over part B and locks the connection (Figure 2e). Figure 2f shows the photo of the joint part with the connector. The assembly completes in 1 min, which we consider sufficiently short. The fixing with the case was experimentally verified to be strong enough; no detachment or leak was observed during the experiments. At the joint, a small ditch at the wall surface will be formed, as shown in Figure 3. Biocompatibility at the ditch will be investigated later.

Since the mechanism is designed such that the blood only flows through the artificial vessels without any deformation, the pressure drop across the connector was negligible, which was later verified in the experiments.

### 2.3. Micro Filtering Device as the Artificial Kidney

The micro filtering device consists of nano porous polyether sulfone (PES) membranes and microfluidic channels made of Titanium (The Nilaco Corporation, Tokyo, Japan). PES membranes are formed by the wet inversion method from PES (Sumitomo Chemical Grade 4800P, Sumitomo Chemical, Co., Ltd., Tokyo, Japan), poly(ethylene glycol) (PEG; molecular weight of 1000, Wako Pure Chemical Industries, Ltd., Osaka, Japan), and N,N-dimethylacetamide (DMAc; Wako Pure Chemical Industries, Ltd.). The microfluidic channels are formed by electrolytic etching. The PES membranes and microfluidic layers are stacked in sequence. The details of the device and the fabrication processes are described elsewhere [8,19,20].

## 3. Experimental Methods

Experiments were conducted based on ISO 10993, the biological evaluation about the medical machine. Thrombus formation and blood coagulation was investigated [21,22,23].

### 3.1. Thrombus Formation Tests In Vitro

**Figure 4:**
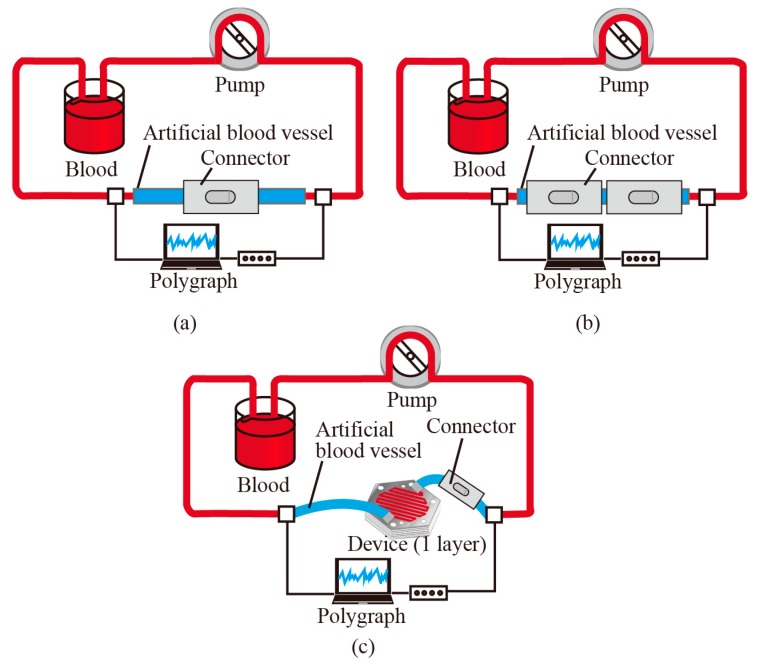
Blood circulation circuit with (**a**) one connector, (**b**) two connectors and (**c**) the connector and the fluidic device. The polygraph measures the pressure before and after the connectors and the device. Blood coagulation at the contact point and change of the activated partial thromboplastin time (APTT) was investigated.

Figure 4a–c illustrates the blood circulation system that includes (a) the connector, (b) two connectors, and (c) the connector and the device, respectively. Thrombus formation at the connection part was investigated in the blood circulation system with one connector (Figure 4a). Human whole blood type A (KOJ, Cosmo Bio, Japan) was used for the experiments. In practical use, the blood flow rate through the connector is expected to be several tens of mL/min. In our prior work, thrombus formation took place in the area where the blood flow rate was low [8]. In this work, since we would like to highlight the thrombus formation and change of blood coagulability, we set the blood flow rate to be 1 mL/min. In case of the setup shown in Figure 4a, the resulting pressure was 90–100 mmHg, which was measured and recorded by polygraph. The fluctuation of the pressure was induced by the pulsation of the pump. The blood contains 3.2% sodium citrate as anti-coagulate and exchanged every 24 h. After 1, 24, 48, and 72 h of circulation, the surface of the artificial blood vessels near the ditch part was treated for cell fixation and then optically investigated with scanning electron microscopy. The cell fixation process included a rinse with PBS, cell fixation with glutaraldehyde solution (2.5% glutaraldehyde, 50% PBS, 47.5% DI water) for 1 d followed by 100% glutaraldehyde for 1 h, rinse with ethanol, and osmium coating for the SEM inspection.

### 3.2. Blood Coagulation Tests In Vitro

Activated partial thromboplastin time (APTT) was measured to assess the blood coagulability caused by the connector. Blood circulation circuits without and with two connectors (Figure 4b) were tested. The flow rate was set to be 1 mL/min and the pressure was 90–120 mmHg. APTT was measured at the beginning and then every 20 min until 120 min.

The APTT measurement protocol is centrifugal extraction of plasma (200 rpm, 11 min), incubation with APTT test reagents for 180 s, and measurement of APTT with calcium chloride solution.

Since the blood contacts air and the quality degrades in 120 min, the in vitro experiments cannot be continued longer than 120 min. In the previous experiment to investigate the thrombus formation, we exchanged the blood, which is not suitable for this experiment. Long term in vivo experiments will be conducted to further validate the effect of the connectors.

### 3.3. Blood Coagulation Tests with the Micro Filtering Device In Vitro

Effect of the micro filtering on the blood coagulation was experimentally investigated. Blood circulation systems including the micro filtering device and both the connector and the micro filtering device (Figure 4c) were prepared. In the experiments, the one-layer filtering device was used. At the beginning, the device is filled with a solution of heparin Na (Mochida Pharmaceutical, Japan) and physiological saline (Otsuka Pharmaceutical, Japan) with a ratio of 1:9 in order to prevent the initial adhesion and coagulation of proteins inside the device. APTT was measured at the beginning and then every 20 min until 120 min (7 data points). APTT measurement protocol is described in Section 3.2. The flow rate was set to be 1 mL/min and the resulting pressure was 90–120 mmHg.

## 4. Experimental Results and Discussion

### 4.1. Thrombus Formation inside the Artificial Blood Vessel

**Figure 5:**
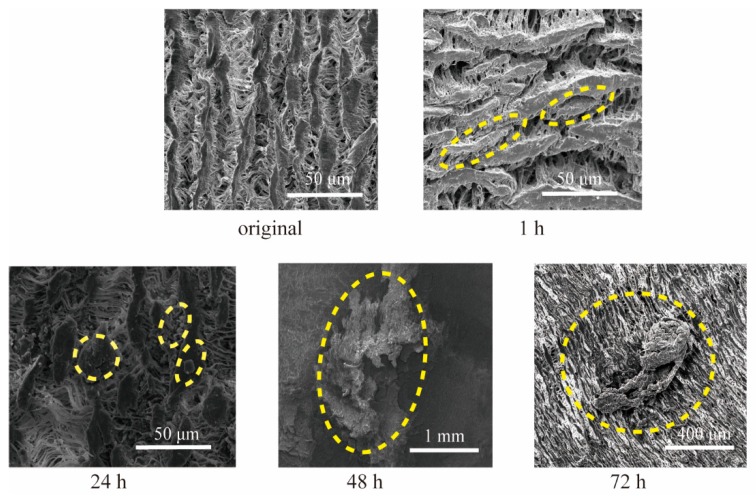
Thrombus formation near the joint observed by SEM. Thrombus formation was observed at each interval. However, it was not concluded that the ditch at the interface initiated the formation.

Figure 5 shows the SEM images of the artificial blood vessel in which blood flowed for 1, 24, 48, 72 h. Aggregates of proteins approximately 50 μm in size were found on the surface after 1 h. After 24 h the number of the aggregates increased and they were found inside the fiber structures. Large aggregates on the order of 1 mm in size were found after 48 h. After 72 h, these large aggregates were still found though the number of them did not increase significantly.

The ditch at the interface of the two artificial blood vessels was suspected to initiate blood coagulation. However, in this experiment the thrombus formation was not limited to the ditch part and no significant effect was found at least for 72 h. It was reported that the thrombosis film was formed on the surface of the artificial vessels, on which vascular endothelial cells subsequently settled. This augments the long-term stability of the artificial vessels [24,25]. We expect the artificial vessels connected with the proposed connector would exploit this phenomenon, though longer-term experiments in vivo will be necessary.

### 4.2. Blood Coagulation Caused by the Connector

**Figure 6:**
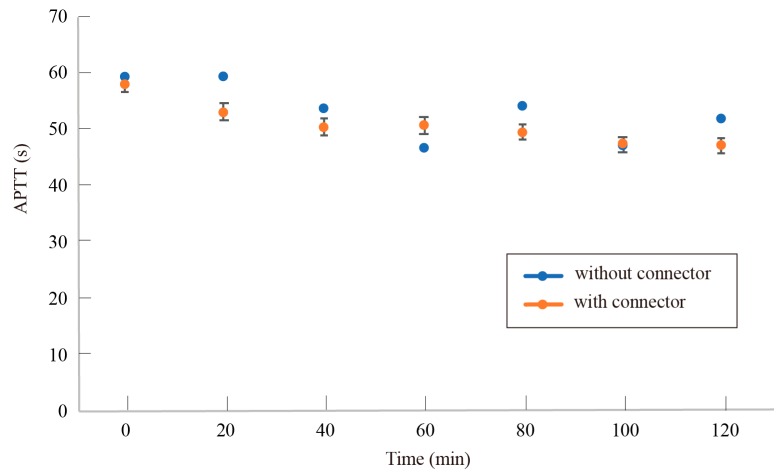
Progress of APTT with and without the connector. No significant differences were induced by the connector during 120 min.

Figure 6 shows the change of APTT with each time interval with or without the connectors. The typical APTT of human blood without anticoagulant is 24–34 s. The APTT was extended to 57–60 s with 3.2% sodium citrate. APTT was found to gradually decrease with time. No significant effect of the connectors was observed for 120 min. This indicates that the proposed connectors do not promote blood coagulation.

Since the quality of blood degrades in 120 min, the in vitro experiments cannot be continued longer than 120 min. Long term in vivo experiments will be conducted to further validate the effect of the connectors.

### 4.3. Blood Coagulation Caused by the Connector and the Filtering Device

**Figure 7:**
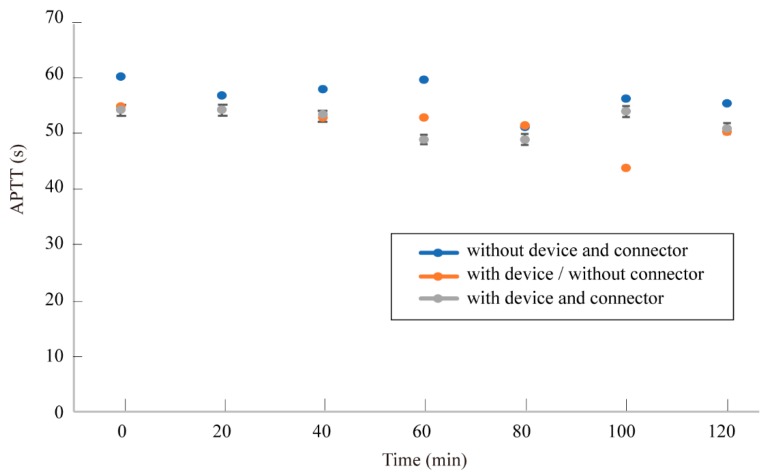
Progress of APTT with the device and the connector. During 120 min of experiments, no significant differences were observed.

Figure 7 shows the variation of APTT with time. For all three cases, i.e., no additional components, with the one-layer micro filtering device, and with the device and the connector, APTT was found to be within the range of 50–60 s.

APTT measurement for 120 min did not show any significant effects of the connectors. The longer experiments need to be conducted to verify the long-term stability of blood coagulability. However, in the in vitro experiments, the property of the blood changes with time and the resulting APTT does not reflect the effect of the connectors. Therefore, long-term in vivo experiments need to be conducted, where control of the anticoagulant concentration in blood will be challenging.

## 5. Conclusions

We designed and demonstrated the enfold connecting system of artificial blood vessels. The design allows blood to contact only the highly biocompatible surfaces of the artificial vessels. Optical investigation after 72 h of blood circulation did not show any significant differences between the joint part (ditch) and the other parts. APTT measurement for 120 min verified that the connector did not augment blood coagulability. The connector proposed herein can be readily applicable to simplify the surgical process of implantable medical devices without degrading the biocompatibility.

## References

[1] Receveur R.A.M., Lindemans F.W., de Rooij N.F. (2007). Microsystem technollgies for implantable applications. J. Micromech. Microeng..

[2] Alici G. (2015). Towards soft robotic devices for site-specific drug delievery. Expert Rev. Med. Devices.

[3] Meng E., Sheybani R. (2014). Insight: Implantable mediclal devices. Lab Chip.

[4] Zahn J.D., Talbot N.H., Liepmann D., Pisano A.P. (2000). Microfabricated polysilicon microneedles for minimally invasive biomedical devices. Biomed. Dev..

[5] Arai M., Kudo Y., Miki N. (2016). Polymer-based candle-shaped microneedle electrodes for electroencephalography on hairy skin. Jpn. J. Appl. Phys..

[6] Miki N., Kanno Y. (2012). Development of a nanotechnology-based dialysis device. Home Dialysis in Japan.

[7] To N., Sanada I., Ito H., Prihandana G.S., Morita S., Kanno Y., Miki N. (2015). Water-permeable dialysis membranes for multi-layered microdialysis system. Front. Bioeng. Biotechnol..

[8] Ota T., To N., Kanno Y., Miki N. (2017). Evaluation of biofouling in stainless microfluidic channels for implantable multilayered dialysis device. Jpn. J. Appl. Phys..

[9] Mineshima M., Kawanishi H., Ase T., Kwasaki T., Tomo T., Nakamoto H. (2018). 2016 update Japanese Society for Dialysis Therapy Standard of fluids for hemodialysis and related therapy. Ren. Replace Ther..

[10] Van der Tol A., Lameire N., Morton R.L., Biesen W.V., Vanholder R. (2019). An international analysis of dialysis services reimbursement. Clin. J. Am. Soc. Nephrol..

[11] Kakisis J.D., Liapis C.D., Breuer C., Sumpio B.E. (2005). Artificial blood vessel: The Holy Grail of peripheral vascular surgery. J. Vasc. Surg..

[12] Byrom M.J., Ng M.K.C., Bannon P.G. (2013). Biomechanics and biocompatibility of the perfect conduit—Can we build one?. Ann. Cardiothorac. Surg..

[13] Ito E., Okano T. (1998). Artificial blood vessels: Structure and property of blood contacting surface. J. Surf. Finish. Soc. Jpn..

[14] Palta S., Saroa R., Palta A. (2014). Overview of the coagulation system. Indian J. Anaesth..

[15] Fogelson A.L., Guy R.D. (2004). Platelet-wall interactions in continuum models of platelet thrombosis: Formulation and numerical solution. Math. Med. Biol..

[16] Basmadjian D. (1990). The effect of flow and mass-transport in thrombogenesis. Ann. Biomed. Eng..

[17] Shinoda T., Arakura H., Katakura M., Shirota T., Nakagawa S. (1990). Usefulness of thrombelastgraphy for dosage monitoring of low molecular weight heparin and unfractionated heparin during hemodialysis. Artif. Organs.

[18] Chen C.C., You J.Y., Ho C.H. (2003). The aPTT assay as a monitor of heparin anticoagulation efficacy in clinical settings. Adv. Ther..

[19] Prihandana G.S., Sanada I., Ito H., Noborisaka M., Kanno Y., Suzuki T., Miki N. (2013). Antithrombogenicity of fluorinated diamond-like carbon films coated nano porous polyethersulfone (PES) membrane. Materials.

[20] Ye G., Miki N. (2009). Multilayered microfilter using PES nano porous membrane applicable as the dialyzer of a wearable artificial kidney. J. Micromech. Microeng..

[21] Weber M., Steinle H., Golombek S., Hann L., Schlensak C., Wendel H.P., Avci-Adali M. (2018). Blood-contacting biomaterials: In vitro evaluation of the hemocompatibility. Front. Bioeng. Biotechnol..

[22] Van Oeveren W., Haan J., Lagerman P., Schoen P. (2002). Comparison of coagulation activity tests in vitro for selected biomaterials. Artif. Organs.

[23] Stang K., Krajewski S., Neumann B., Kurz J., Post M., Stoppelkamp S., Fennrich S., Avci-Adali M., Armbruster D., Schlensak C. (2014). Hemocompatibility testing according to ISO 10993-4: Discrimination between pyrogen-and device-induced hemostatic activation. Mater. Sci. Eng. C.

[24] Noishiki N. (1998). A concept of tissue engineering in the development of small diameter vascular prothesis. Artif. Organ..

[25] Xue L., Greisler H.P. (2003). Biomaterials in the development and future of vascular grafts. J. Vasc. Surg..

